# Supplementary material for: Complexome profiling of the Chlamydomonas psb28 mutant reveals TEF5 as an early PSII assembly factor
Source: Plant Cell. 2025 Mar 17;37(6):koaf055. doi: 10.1093/plcell/koaf055 (PMC12164590; doi:10.1093/plcell/koaf055)
Supplement: koaf055_Supplementary_Data [file koaf055_supplementary_data.zip › psb28_interactive.zip!/plots/intensity/qualityPlots/peptides/VIPP1_Cre13.g583550.t1.2.html]

### Cre13.g583550.t1.2

Trivial name: VIPP1  
SeqCoverage [median]: 24.30  
Mass: 31.61 kDa  
  
 

| Sequence | MissedCleavage | Mass [Da] | wtIntensity | muIntensity | Score |
| --- | --- | --- | --- | --- | --- |
| GMLPSTTSAVGSLPEPR | 0 | 1698.86 | 330.15 | 2093.07 | 113.99 |
| MLVGSDTIDNK | 0 | 1191.58 | 0.00 | 526.29 | 69.60 |
| MLVGSDTIDNKFK | 1 | 1466.74 | 0.00 | 583.62 | 74.76 |
| TYQEQADQLK | 0 | 1222.58 | 234.10 | 488.95 | 136.57 |
| VQVDQLSGASGDVLNNTR | 0 | 1871.93 | 0.00 | 1731.67 | 121.18 |
|  |  |  | 564.3 | 5423.6 |  |
